# Generating coherent and ultrashort X-ray pulses via HHG-seeding in storage rings

**DOI:** 10.1107/S1600577521013382

**Published:** 2022-01-19

**Authors:** Yujie Lu, Chao Feng, Lingjun Tu, Changliang Li, Bocheng Jiang, Dong Wang

**Affiliations:** a Shanghai Institute of Applied Physics, Chinese Academy of Sciences, Shanghai 201800, People’s Republic of China; b School of Physical Science and Technology, ShanghaiTech University, Shanghai 201210, People’s Republic of China; c Shanghai Advanced Research Institute, Chinese Academy of Sciences, Shanghai 201210, People’s Republic of China; d University of Chinese Academy of Sciences, Beijing 100049, People’s Republic of China

**Keywords:** high harmonic generation, angular dispersion modulation, storage rings, ultrashort, free-electron lasers

## Abstract

The generation of fully coherent and femtosecond time-scale radiation pulses in the X-ray regime is one of the most common demands of ring-based synchrotron light source users. In this paper, a method that utilizes the recent proposed angular dispersion induced microbunching technique to convert external light from high-harmonic generation (HHG) to coherent light at shorter wavelength is proposed. Numerical simulations using the practical parameters of a diffraction-limited storage ring demonstrate the generation of coherent pulse trains with photon energy as high as 2 keV, pulse duration as short as ∼10 fs and high peak brightness directly from an HHG source at 13 nm.

## Abstract 2

A method that utilizes the recent external light from high-harmonic generation (HHG) to coherent light at shorter wavelength is proposed. Numerical simulations with parameters of a diffraction-limited storage ring demonstrate the generation of coherent pulse trains with photon energy as high as 2 keV, pulse duration as short as ∼10 fs and high peak brightness directly from an HHG source at 13 nm.

## 1. Introduction

Over the past years, synchrotron light sources have supported plenty of brilliant X-ray capabilities that are beneficial to many disciplines (Zhao, 2010 ▸), such as physics, chemistry, biology and material science, *etc*. Ring-based light sources have the advantages of high repetition rate and simultaneous operation of multiple beamlines. In the past decades, we have seen the development of the linac-driven free-electron lasers (FELs), which push the peak brilliance to an unprecedented level, up to about ten orders of magnitude higher than that of synchrotron radiation sources. Full transverse and longitudinal coherence are presently achievable with external seeded FELs in the EUV and soft X-ray wavelength range (Demidovich *et al.*, 2012 ▸; Allaria *et al.*, 2012 ▸, 2013 ▸; Yu *et al.*, 2019 ▸; Ribič *et al.*, 2019 ▸; Feng *et al.*, 2019 ▸). Merging these two techniques can make up for their own disadvantages and open up the possibility of developing light sources with unique properties that are of remarkable interest to light source researchers and users.

Borrowing the idea of electron beam manipulation with external lasers in seeded FELs, several techniques have been developed to enhance the performances of storage-ring-based light sources (Zholents & Zolotorev, 1996 ▸; Girard *et al.*, 1984 ▸; Yu, 1991 ▸; Yu *et al.*, 2000 ▸; Xiang & Stupakov, 2009 ▸; Evain *et al.*, 2012 ▸; Feng *et al.*, 2014 ▸; Xiang & Wan, 2010 ▸). However, these techniques still require large energy modulation amplitudes to reach the soft X-ray region. To overcome this problem, the angular-dispersion-induced microbunching (ADM) technique has been proposed to modulate the beam angular divergence (Feng & Zhao, 2017 ▸; Wang *et al.*, 2019 ▸), which requires a very low power seed laser with a relatively simple lattice design. This scheme makes full use of the advantage of low vertical emittance in the storage rings and, if the parameters are chosen properly, sharp microbunches can be generated. This technique is very promising for generating intense radiation pulses at wavelengths as short as several nanometres. However, limited by the wavelength of the seed laser, further extending the short-wavelength coverage of this technique is still very challenging.

The short-wavelength seed laser can be obtained through the high harmonic generation (HHG) technique (McNeil *et al.*, 2007 ▸; Springate & Tisch, 2011 ▸; Maltezopoulos *et al.*, 2014 ▸; Wu *et al.*, 2007*a* ▸,*b* ▸; Kanda *et al.*, 2020 ▸; Willner *et al.*, 2011 ▸; Dunning *et al.*, 2011 ▸). HHG is produced by focusing a short laser pulse onto a gas-jet and radiating the high harmonic of the laser whose wavelength is much shorter than the initiate laser. So far, many groups have made efforts to achieve high-power FELs by directly amplifying the EUV pulse from HHG (McPherson *et al.*, 1986 ▸; Ditmire *et al.*, 1995 ▸; Takahashi *et al.*, 2004 ▸; Zhang *et al.*, 2018 ▸; Lou *et al.*, 2019 ▸). However, this still requires major progress in laser technology to enhance the output peak power of HHG to satisfy the requirements of seeding a FEL for HHG. In this paper, we proposed to apply HHG as the seed laser to the ADM. This proposal takes full advantages of HHG with short wavelength and ADM with low demand on the seed laser power. With characteristic ideal beam parameters based on diffraction-limited storage rings (DLSRs), we show that coherent radiation pulses with photon energy as high as 2 keV, pulse duration as short as ∼10 fs and high peak brightness can be produced in storage rings. This kind of light source may open up new research directions for ultrafast science in storage rings.

## 2. Schematic layout

**Figure 1:**
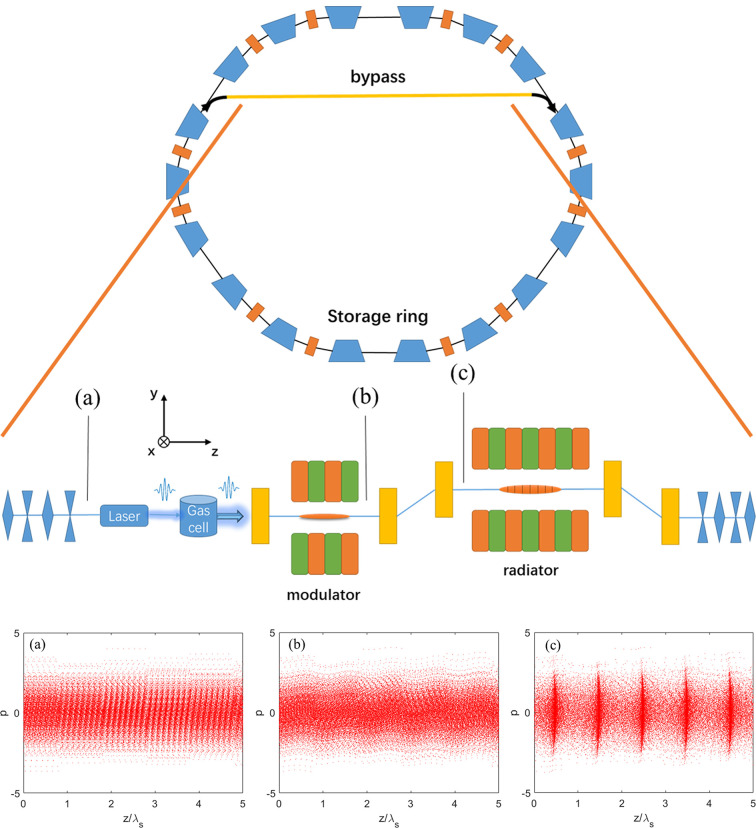
Schematic layout of a storage ring with a bypass line showing the proposed technique and the longitudinal phase space evolution: (*a*) the initial phase space; (*b*) the phase space at the entrance of the dispersion section; (*c*) the phase space at the exit of the dispersion section.

**Figure 2:**
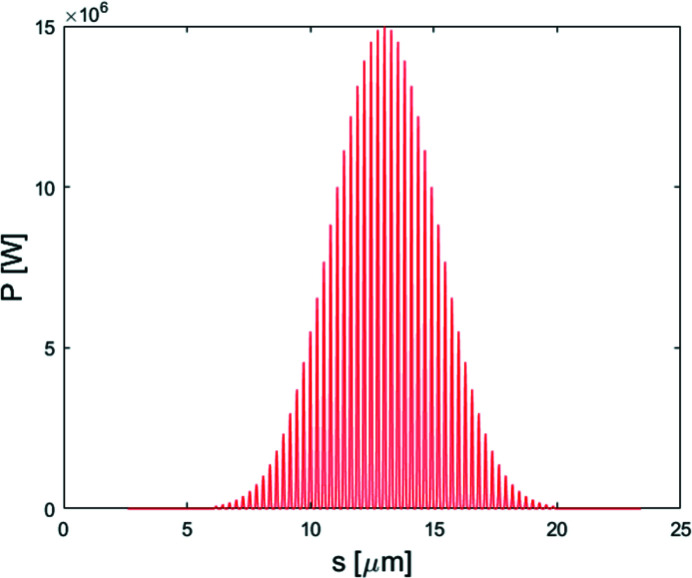
Longitudinal profile distribution of the HHG seed laser.

In the proposed scheme, the electron beam first passes through a magnetic bend to generate angular dispersion. After that, an HHG source is used to interact with the electron beam in the undulator resonant to the seed wavelength to generate energy modulation. The bunching factor of ADM is mainly determined by the initial angular divergence; therefore, the energy modulation amplitude can be much smaller than the initial uncorrelated energy spread of the electron beam, as shown in Fig. 1 ▸(*b*). The wavelength of HHG is chosen to be 13 nm and the peak power is about 15 MW (Takahashi *et al.*, 2004 ▸; Dunning *et al.*, 2011 ▸). The pulse duration is about 15 fs whereas the sub-pulse duration is only ∼200 as. Although the repetition rate of the drive laser with an energy of mJ magnitude is just in the multi-kHz range, utilizing intracavity HHG in mode-locked oscillators is possible in order to reach a MHz-level repetition rate (Kanda *et al.*, 2020 ▸). Here we choose a repetition rate of 1 kHz for the HHG seed (Dunning *et al.*, 2011 ▸; Ding *et al.*, 2014 ▸; Springate & Tisch, 2011 ▸; Willner *et al.*, 2011 ▸). The average power of the seed is 60 µW. The photon energy range of HHG-seeding is 50–100 eV. In this regime, the laser intensity is kept to 4 × 10^14^ W cm^−2^. The 60 m path interferometer between the two beams has been shown to have 16 fs r.m.s. stability in the Central Laser Facility (Hooker *et al.*, 2009 ▸). According to Springate & Tisch (2011 ▸), the tolerable jitter on the laser beam position and pointing were <10 µm and <3 µrad for power fluctuations of <1% which is acceptable. It is promising that the above specifications can be met. In brief, this kind of HHG is quite suitable for seeding the ADM. As shown in Fig. 2 ▸, the fundamental longitudinal structure of HHG seed can be written as 

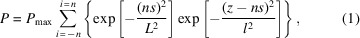

where *P*_max_ is the peak power of the seed laser, *z* is the longitudinal position of the light field, *n* is the number of sub-pulses, *s* is the space between the sub-pulses, *L* is the duration of the pulse envelope and *l* is the duration of the sub-pulse. The electron beam is then sent through the dogleg to generate a density modulation. Taking advantage of the low vertical emittance in the storage rings, there is strong microbunching in the longitudinal phase space which means high harmonic frequency can be achieved, as shown in Fig. 1 ▸(*c*). However, a large vertical beta function in the proposed scheme is needed, with the result that beam lifetime will be shortened and the linear optics will be very sensitive to the field errors in a storage ring. Therefore, we propose to implement the proposed scheme in a bypass (Murphy & Pellegrini, 1985 ▸; Nuhn *et al.*, 1992 ▸; Di Mitri & Cornacchia, 2015 ▸; Lee, 2019 ▸; Li *et al.*, 2021 ▸), as shown in Fig. 1 ▸. Before the electron beam is kicked into the bypass, it circulates in the storage ring without passing through the proposed scheme and stays at the equilibrium status. After passing through the proposed scheme, the beam is kicked back into the storage ring for damping. After the damping, the initial status of the electron beam will be erased and the beam can be kicked back into the bypass again. A realistic storage ring design with multiple turns simulation based on the ADM has been presented by Li *et al.* (2020 ▸). The optimized storage ring design with a circumference of 900 m was demonstrated by numerical simulations to obtain a repetition rate of 10 kHz. In this work, we mainly focus on converting external light from HHG to coherent light at shorter wavelength through the ADM technique.

## 3. Simulations

The theoretical derivation of ADM is given by Feng & Zhao (2017 ▸). Here we only show the simulation results. Three-dimensional simulations are employed to illustrate the performance of the proposed scheme. The processes of energy modulation and FEL lasing are simulated using *GENESIS* (Reiche, 1999 ▸). The transmission processes of the electron beam through the bend magnet and dogleg are simulated by *ELEGANT* (Borland, 2001 ▸) with second-order transport effects taken into account. Typical parameters of a DLSR (Bai *et al.*, 2021 ▸), as summarized in Table 1 ▸, are chosen to perform these simulations. The initial r.m.s. beam size is σ_*x*,*y*_ = 25 µm at the entrance of ADM. The bunching factor of ADM is given by (Feng & Zhao, 2017 ▸) 

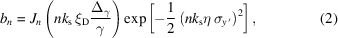

where *J*_*n*_ is the *n*th-order Bessel function, *k*_s_ is the seed laser wavenumber, ξ_D_ is the momentum compaction of the dogleg, Δ_γ_ is the energy modulation amplitude, γ is the relativistic parameters of the electron beam energy, η is the dispersion and 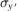 is the initial angular divergence of the electron beam.

**Figure 3:**
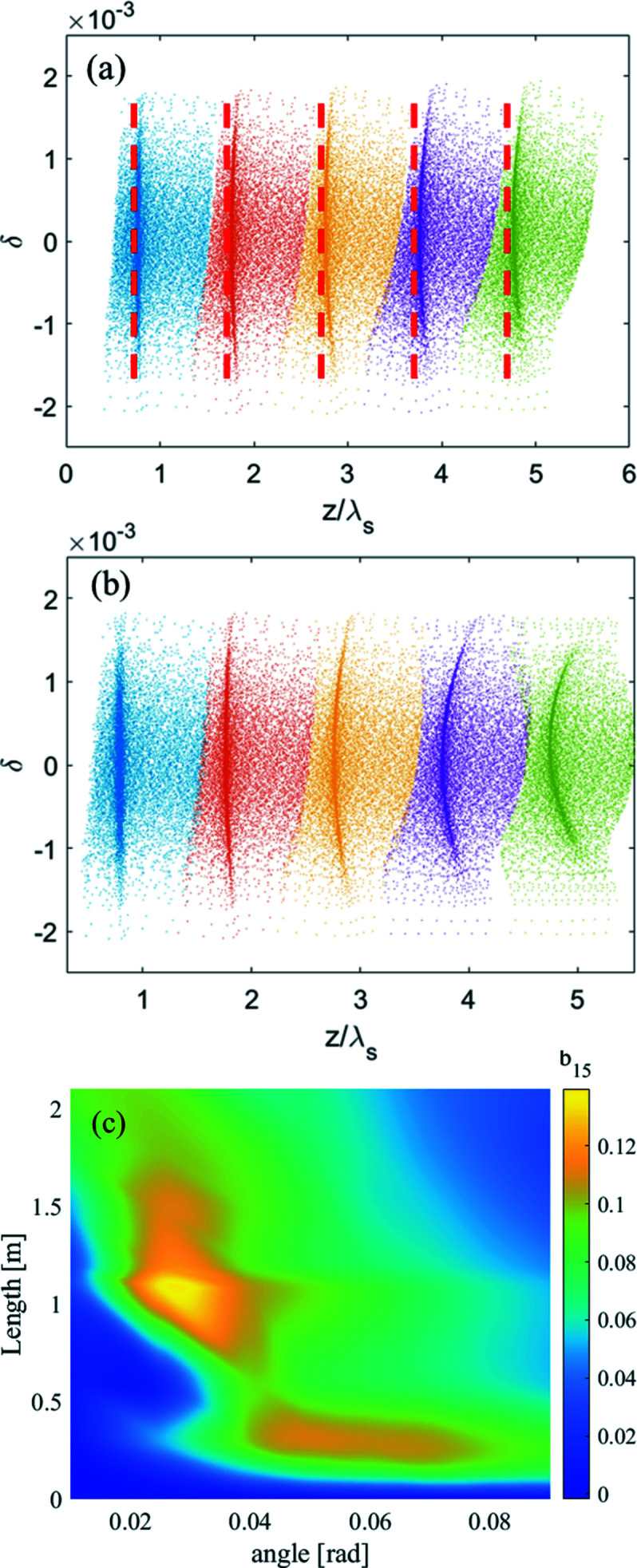
(*a*) The longitudinal phase space (blue, orange, yellow, purple, green) at the exit of the dispersion section with different modulator lengths (0.6 m, 1.2 m, 1.8 m, 2.4 m, 3 m) and the same bend angle (30 mrad). (*b*) The longitudinal phase space (blue, orange, yellow, purple, green) at the exit of the dispersion section with different strengths (10 mrad, 30 mrad, 50 mrad, 70 mrad, 90 mrad) of the first dipole and the same modulator length (1 m). (*c*) The peak bunching factor distribution of the 15th harmonic with different strength of the dipole and different modulator length.

According to the optimized conditions 1 + *h*ξ_D_ = 0 and ξ_D_ = η*t* (Feng & Zhao, 2017 ▸), where *h* is the energy chirp induced by the laser-beam interaction and *t* is the bending angle of the dipole, low-energy modulation amplitude in the proposed scheme results in a large ξ_D_. To obtain a small enough η and then enlarge the bunching factor [equation (2)], it is necessary to increase the strength of the first dipole to a large value in the proposed scheme. The divergence of the electron beam after the angular modulation can be written as 

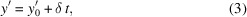

where 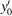 is the initial divergence of the electron and δ is the relative energy deviation with respect to the reference particle. Because of the relatively large energy spread of the beam in DLSRs, the divergence will rise tremendously with the increase of bending angle of the dipole which means that the horizontal velocity differences of the particles become more obvious. Therefore, when the electron beam passes through the modulator, the particles with larger horizontal velocities will slip in phase with respect to the particles with lower horizontal velocities. As shown in Figs. 3 ▸(*a*) and 3(*b*), the produced microbunch evolves into a curved shape with the increase of the modulator length and bending angle of the dipole. The peak bunching factor distribution with the change of modulator length and dipole magnet strength is shown in Fig. 3 ▸(*c*). Due to the second-order transport effect, the peak bunching factor of the 15th harmonic begins to descend when the bending angle is greater than 25 mrad and the length of the modulator is longer than 1 m as shown in Fig. 3 ▸(*c*).

**Figure 4:**
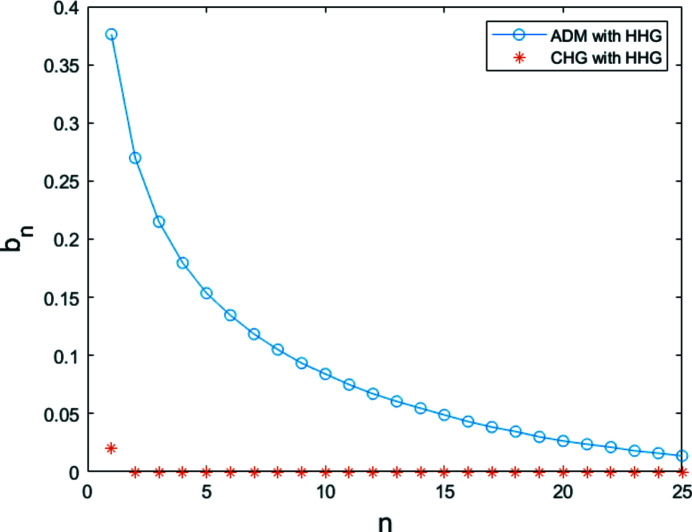
Comparison between the bunching factors of ADM and CHG with HHG as the seed laser.

**Figure 5:**
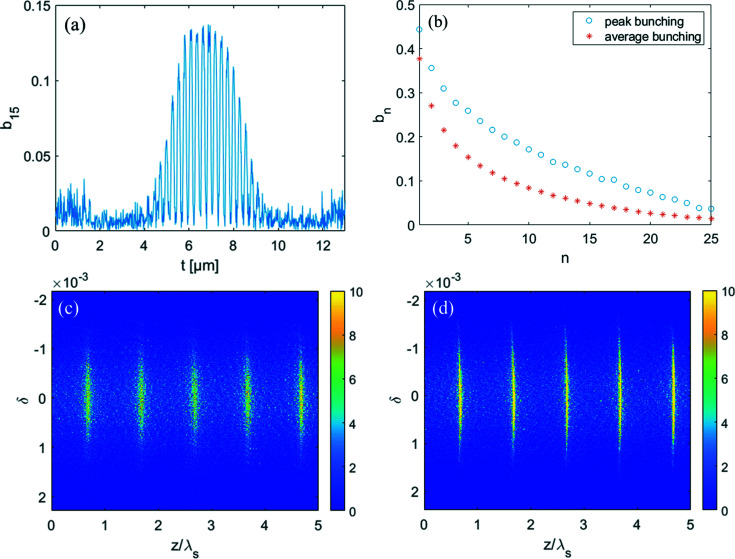
(*a*) The 15th bunching factor distribution at the entrance of the radiator. (*b*) Comparison of the bunching factor of the proposed scheme. The blue circles are the results of peak bunching factors. The red asterisks are the results of average bunching factors. (*c*) Longitudinal phase space with relatively small energy modulation. (*d*) Longitudinal phase space with relatively large energy modulation.

After passing through a dipole with a bending angle of 25 mrad and length of 0.5 m, the electron beam is sent into a short modulator with period length of 30 mm and period number of 34 to interact with an HHG seed at 13 nm. The longitudinal profile of the HHG seed is shown in Fig. 2 ▸. The dipole magnet in the dispersion section has a bending angle of approximately 30 mrad. The transverse dispersion strength is about 1.34 mm and the momentum compaction factor is about 34 µm. Fig. 4 ▸ shows the bunching factor at various harmonic numbers for ADM and conventional coherent harmonic generation (CHG) (Yu, 1991 ▸) via HHG seeding. Due to the low power of the HHG seed, one can obtain a bunching factor of only 2% at the fundamental wavelength (13 nm) for the conventional CHG. The local bunching factor distribution of the proposed scheme at the 15th harmonic is shown in Fig. 5 ▸(*a*) and follows the longitudinal profile of the HHG seeding. The bunching factor at the 15th harmonic is close to 15%. As shown in Fig. 5 ▸(*b*), one can find that the maximal bunching factor achievable with the HHG seed is much larger than the average bunching factor based on numerical simulation due to the pulse-train distribution of the seed laser. Fig. 5 ▸ also presents the formed micro-bunching at different locations of the electron beam, *i.e.* modulations with relatively low power [Fig. 5 ▸(*c*)] and high power [Fig. 5 ▸(*d*)].

**Figure 6:**
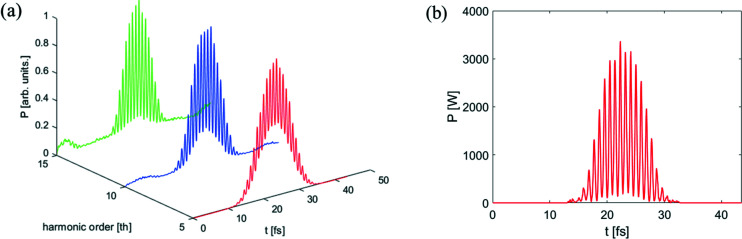
(*a*) Normalized radiation pulses at different harmonics from a 3 m-long radiator. (*b*) Longitudinal profile of the radiation pulse train at the 15th harmonic from a 2.5 m-long radiator.

**Figure 7:**
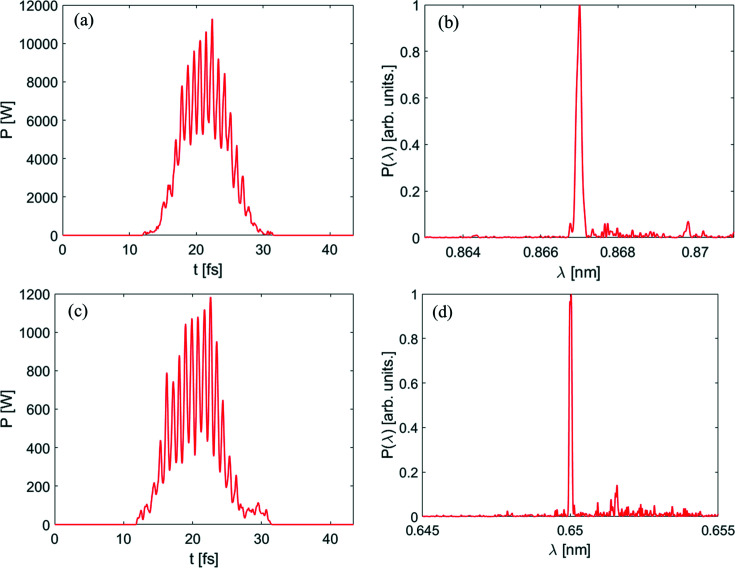
Performances of the proposed technique at the 15th and 20th harmonics of the seed. Panels (*a*) and (*c*) are the output radiation pulses at the 15th and 20th harmonics at the exit of the radiator. Panels (*b*) and (*d*) represent the spectra at the 15th and 20th harmonics at the exit of the radiator.

**Figure 8:**
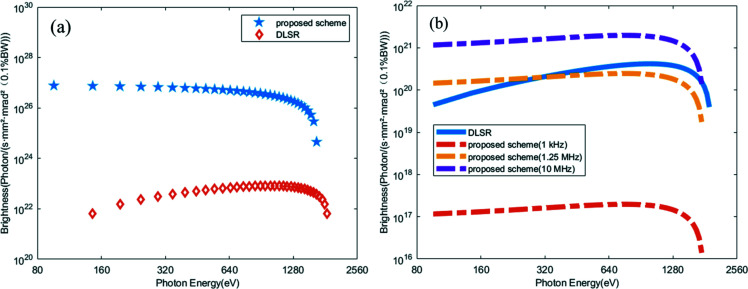
(*a*) Comparison of the peak brightness between the proposed scheme and diffraction-limited storage ring. (*b*) Comparison of the average brightness for the diffraction-limited storage ring (100 MHz) and the proposed scheme (1 kHz, 1.25 MHz, 10 MHz) without considering the limitation of the laser repetition rate.

After passing through the dogleg, the electron beam is sent into the radiator to generate coherent radiation pulses at shorter wavelength. The simulation results are summarized in Figs. 6 ▸ and 7 ▸. Output radiation pulses at *z* = 3 m in the radiator are shown in Fig. 6 ▸(*a*). Viewed in the figure, the relative slippage between radiation and electron beam is longer than the spacing between the spikes so that the attosecond structure is gradually washed out with the decrease of radiation wavelength. As shown in Fig. 6 ▸(*b*), the peak power of the radiation pulse train at the 15th harmonic (0.86 nm) is about 3.2 kW at *z* = 2.5 m in the radiator. A possible application of this attosecond pulse train as a kicking field in the kicker rotor has been theoretically examined for the first time (Mašović, 2021 ▸). Due to the slippage effect, the duration of each spike increases from the original ∼200 as (FWHM) to ∼300 as (FWHM). Fig. 7 ▸ represents the fully coherent radiation properties at the 15th and 20th harmonic (∼2 keV) of the seed. From Fig. 7 ▸(*a*), the maximum output peak power at the 15th harmonic (0.86 nm) is close to 12 kW. The output spectral bandwidth is about 0.18%, which is about 1.32 times the Fourier-transform limit. A fully coherent radiation pulse with photon energy as high as 2 keV can also be achieved, as shown in Figs. 7 ▸(*c*) and 7(*d*). According to Takahashi *et al.* (2004 ▸) and Springate & Tisch (2011 ▸), continuous tunability is achieved by varying the wavelength of the drive laser over ±25 nm from the 800 nm center wavelength. The HHG seed is designed to cover the energy range from 50 to 100 eV (32 to 13 nm). In our proposed scheme, the radiation wavelength based on the ADM via HHG seeding can be turned from 32 nm to 0.65 nm (50–2000 eV). The brightnesses for a normal DLSR and for the proposed technique are also calculated. The peak brightness achievable with our proposed method is about four to five orders of magnitude higher than the normal DLSR with the same electron beam parameters, as shown in Fig. 8 ▸(*a*). Fig. 8 ▸(*b*) shows the calculation results of the average brightnesses. Limited by the repetition rate (1 kHz) of the HHG seed, the average brightness of the proposed method is three to four orders of magnitude lower than that of a normal DLSR with a repetition rate of 100 MHz, as shown in Fig. 8 ▸(*b*). The relativistic electron bunches stored in a storage ring through some manipulation techniques at the femtoseond scale (∼100 fs) can reach a MHz repetition rate (Abo-Bakr *et al.*, 2003 ▸; Holldack *et al.*, 2005 ▸; Prigent *et al.*, 2013 ▸; Schoenlein *et al.*, 2000*a* ▸,*b* ▸; Zholents *et al.*, 1999 ▸; Hwang *et al.*, 2020 ▸). In the proposed scheme, the electron beam was modulated by the seed laser with duration of only 15 fs, which is much shorter than that destroyed by the femtoslicing technique. Therefore, it is promising to reach the 1–10 MHz level with the proposed technique without considering the limitation of the laser repetition rate. If the repetition rate of the utilized HHG seed can be increased to as high as MHz magnitude in accordance with the bunches using technologies like mode-locked oscillators (Kanda *et al.*, 2020 ▸) in the future, the average brightness of the proposed scheme can be promisingly comparable with and even higher than that of a normal DLSR, as shown in Fig. 8 ▸(*b*).

## 4. Conclusion

We propose to utilize the recently proposed ADM scheme to convert an HHG seed to shorter wavelength in a DLSR. An HHG source with peak power of 15 MW and single-pulse energy of 60 nJ is utilized to modulate the electron beam. Taking advantage of ADM and the characteristics of the DLSR, fully coherent X-ray pulses with high peak brightness and pulse duration as short as 9 fs in the soft X-ray region can be generated through a 16 m-long undulator. The wavelength of the fully coherent ultrafast radiation based on a storage ring is pushed to the sub-nanometre range for the first time. It can be used to perform short-wavelength, high-repetition pump–probe experiments and nonlinear X-ray optics experiments that were previously impossible based on storage rings. Besides, we also compared the radiation brightness between a normal DSLR and a DSLR with the proposed technique. The peak brightness of the proposed scheme is much higher than for the normal DLSR. The average brightness of the proposed scheme is constrained by the repetition rate of the seed laser. Utilizing intracavity HHG in a mode-locked oscillator is possible in order to reach a MHz-level repetition rate (Kanda *et al.*, 2020 ▸). The repetition rate of the radiation pulse can be increased in the storage ring through this technique, thereby improving the average brightness. In addition, due to the huge relative energy spread in the storage ring, the large strength of the first bend magnet may cause large non-linear effects in the modulator which will affect the performance of the proposed scheme. Further studies on these topics are still ongoing.

## Figures and Tables

**Table 1:** Nominal electron beam parameters used in the simulation

Beam energy	2 GeV
Relative energy spread	0.06%
Peak current	20 A
Geometric horizontal emittance	0.2 nm rad
Geometric vertical emittance	2 pm rad
